# Postbiotics in colorectal cancer: intervention mechanisms and perspectives

**DOI:** 10.3389/fmicb.2024.1360225

**Published:** 2024-02-21

**Authors:** Wei Xie, Yu-Sen Zhong, Xue-Jian Li, You-Kun Kang, Qian-Yu Peng, Hua-Zhong Ying

**Affiliations:** Key Laboratory of Experimental Animal and Safety Evaluation, Hangzhou Medical College, Hangzhou, China

**Keywords:** postbiotics, colorectal cancer, intestinal microbiota, metabolites, intestinal immunity

## Abstract

Colorectal cancer (CRC) is a common malignancy affecting the gastrointestinal tract worldwide. The etiology and progression of CRC are related to factors such as environmental influences, dietary structure, and genetic susceptibility. Intestinal microbiota can influence the integrity of the intestinal mucosal barrier and modulate intestinal immunity by secreting various metabolites. Dysbiosis of the intestinal microbiota can affect the metabolites of the microbial, leading to the accumulation of toxic metabolites, which can trigger chronic inflammation or DNA damage and ultimately lead to cellular carcinogenesis and the development of CRC. Postbiotics are preparations of inanimate microorganisms or their components that are beneficial to the health of the host, with the main components including bacterial components (e.g., exopolysaccharides, teichoic acids, surface layer protein) and metabolites (e.g., short-chain fatty acids, tryptophan metabolite, bile acids, vitamins and enzymes). Compared with traditional probiotics, it has a more stable chemical structure and higher safety. In recent years, it has been demonstrated that postbiotics are involved in regulating intestinal microecology and improving the progression of CRC, which provides new ideas for the prevention and diagnosis of CRC. In this article, we review the changes in intestinal microbiota in different states of the gut and the mechanisms of anti-tumor activity of postbiotic-related components, and discuss the potential significance of postbiotics in the diagnosis and treatment of CRC. This reviews the changes and pathogenesis of intestinal microbiota in the development of CRC, and summarizes the relevant mechanisms of postbiotics in resisting the development of CRC in recent years, as well as the advantages and limitations of postbiotics in the treatment process of CRC.

## 1 Introduction

Colorectal cancer (CRC) is a common malignant tumor of the gastrointestinal tract, ranking third worldwide in terms of both incidence and mortality rates (Bray et al., 2018). The etiology and progression of CRC is influenced by genetic factors, chronic inflammatory bowel disease, dietary habits, and environmental factors (Lund et al., 2011). The intestinal microbiota, as an important component of the gut, has been increasingly demonstrated to be involved in the development of CRC. The intestinal microbiota is a complex community of bacteria, fungi, archaea, protozoa, and viruses (Costello et al., 2012). The intestinal microbiota plays a crucial role in the host’s nutritional, metabolic, and immune functions, as well as other physiological processes by secreting a variety of metabolites (Dzutsev et al., 2015). Dysbiosis of intestinal microbiota is often manifested as a decrease in beneficial bacteria and an increase in harmful bacteria, and the disruption of this balance will result in the disturbance of microbial metabolites, leading to the accumulation of toxic metabolites, which in turn will lead to the destruction of the intestinal mucosal barrier, resulting in chronic inflammation and DNA damage, and ultimately triggering the cellular carcinogenesis and the development of CRC (Clinger and Hao, 2021).

Probiotics are defined as “live, non-pathogenic microorganisms that, when given in sufficient amounts, can be beneficial to the health of the host” and are mainly Bifidobacteria, *Lactobacillus* and other acid-producing bacteria, including *Streptococcus*, *Enterococcus*, and *Lactococcus*. Probiotics can play a role in maintaining a healthy intestinal microbiota, preventing the invasion of pathogenic microorganisms, and stabilizing and strengthening the intestinal barrier function through the secretion of anticancer or anti-inflammatory substances, such as short-chain fatty acids (SCFAs), vitamins K or B, and others. Prebiotics are mostly composed of non-starch polysaccharides and oligosaccharides that are difficult to be digested by enzymes in the body, which can provide nutritional support for beneficial bacteria in the intestinal tract, such as resistant starch, lactulose, inulin, oligofructose, oligogalactose and so on. And synbiotics are a mixture of probiotics and prebiotics, which can play the roles of both probiotics and prebiotics at the same time. NGP can be used as a preventive and therapeutic potential application for CRC, in contrast to traditional probiotics, which are based on macrogenomics studies that analyze microbiota differences between healthy and diseased individuals, identify live microorganisms and administer them in a strain-specific and dose-dependent manner, resulting in health benefits for the host (Martín and Langella, 2019). In addition, FMT is the most direct way to regulate the intestinal microbiota, and FMT can intervene and treat intestinal diseases by proposing specific flora from the flora of healthy donors, mating and culturing them and colonizing them in the patient’s intestinal tract to rebuild the patient’s intestinal microcosm, thus achieving intervention and treatment of intestinal diseases. More and more studies now show that oral administration of probiotics, NGP, or FMT can restore the balance of intestinal microbiota and thus achieve improvement in CRC progression (Eslami et al., 2019; Chen et al., 2022). However, being live microorganisms and the uncertainty of their growth during processing, there are potential biosafety risks associated with the use of live bacteria as a therapeutic strategy for CRC.

Postbiotics are preparations of inanimate microorganisms or their components that are beneficial to the health of the host, with the main components including bacterial components and metabolites. As a new type of biological agent, Postbiotic shows good benefits in regulating the balance of intestinal microbiota (Guéniche et al., 2010). It has a relatively stable chemical structure and higher biosafety than traditional probiotics, NGP and FMT. In recent years, there has been increasing evidence that oral administration of postbiotics can regulate intestinal microbiota, improve immunity and reduce the incidence of diarrhea (Yeom et al., 2021; Jung et al., 2023). Therefore, postbiotics has been emphasized as a complementary therapeutic strategy for CRC. This reviews the changes and pathogenesis of intestinal microbiota in the development of CRC, and summarizes the relevant mechanisms of postbiotics in resisting the development of CRC in recent years, and discusses the advantages and limitations of postbiotics in the treatment process of CRC.

## 2 The role of intestinal microbiota dysbiosis in the development of CRC

The human body harbors a wide variety of microorganisms, primarily located in the oral cavity, intestines, respiratory tract, skin, vagina, and other mucosal surfaces, creating a highly intricate microbial ecosystem. The intestinal tract, in particular, hosts a significant population of commensal bacteria, making it the largest reservoir of commensal microbiota. This bacterial community comprises approximately 800 species, collectively weighing around 1 to 2 kilograms (Zhang et al., 2018). The intestinal tract is considered the most abundant, diverse, and functionally significant microbial community in the human body (Meng et al., 2018). The intestinal microbiota demonstrates characteristics such as diversity, stability, resistance to drugs and antiretrovirals, and plays a crucial role in maintaining normal physiological functions and disease development. Dysbiosis of intestinal microbiota is primarily linked to changes in bacterial diversity, which leads to the proliferation of harmful bacteria in the gut. This can result in the release of virulence factors, suppression of the immune system, and stimulation of inflammation, ultimately contributing to the onset of CRC.

**FIGURE 1:**
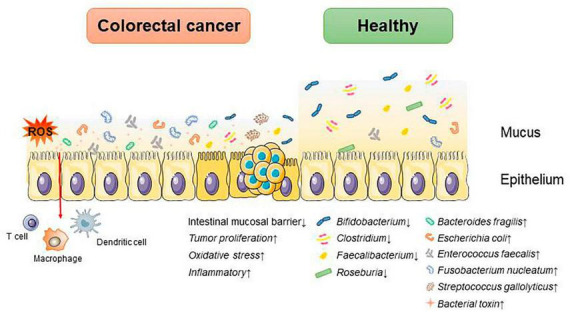
Dysbiosis of intestinal microbiota accelerates the progression of CRC. An increase in associated pathogenic bacteria promotes CRC progression by disrupting the intestinal mucosal barrier, triggering inflammatory responses and oxidative stress, and altering the tumor proliferation cycle.

Microorganisms parasitizing the human gut are classified in the phylum *Bacteroidetes*, *Firmicutes*, *Proteobacteria*, *Fusobacteria*, *Actinobacteria*, *Verrucomicrobia*, and *Spirochaetes*. Studies have shown that the composition of the intestinal microbiota differs between the healthy and CRC states, and these changes are summarized in Figure 1 (Kvakova et al., 2021).

Under normal conditions, *Anaplasma*, *Bifidobacterium*, *Lactobacillus*, *Streptococcus*, *Enterococcus*, *Clostridium*, and *Escherichia coli* are the predominant species in the human intestinal tract, which participate in the formation of the intestinal mucosal barrier to inhibit the growth and invasion of harmful bacteria, and at the same time, they secrete metabolites, such as short-chain fatty acids, indole, etc., which affect the intestinal immunity and participate in the metabolism of the human body (Hooper and Gordon, 2001).

Whereas in disease states it is often characterized by a decrease in beneficial bacteria such as *Bifidobacterium*, *Lactobacillus*, and *Bacteroides* and an increase in hamful bacteria such as enterotoxin producing *Bacteroides*, *Escherichia coli*, and *Clostridium difficile*. A study suggests that intestinal microbial species are strongly associated with the development of CRC, showing increased abundance of pro-inflammatory opportunistic bacteria such as *Bacteroides fragilis*, *Escherichia coli*, *Enterococcus faecalis*, *Fusobacterium nucleatum*, and *Streptococcus gallolyticus* in patients with CRC. Meanwhile, the abundances of *Bifidobacterium*, *Clostridium*, *Faecalibacterium*, and *Roseburia declined* (Janney et al., 2020). The mechanisms by which intestinal microbiota affects CRC progression include destroying the intestinal mucosal barrier; regulating the cell cycle of CRC tumor cells, promoting CRC proliferation and metabolism; reprogramming the tumor immune microenvironment; causing DNA damage; triggering inflammatory responses; inducing gene mutations and altering the resistance to tumor chemotherapy, and so on. These mechanisms are summarized in Table 1.

**TABLE 1:** Mechanisms associated with CRC progression induced by intestinal microbes.

Organism	The role for CRC	Mechanism	References
*Fusobacterium nucleatum*	Promote	• Regulates the E-cadherin/β-catenin signaling pathway and promotes tumor cell proliferation. • Activation of NF-κB pathway/RAS-MAPK pathway via MYD88 enhances CRC cell proliferation. • Increased H3K27ac histone modification in CRC cells, which activates glycolysis and carcinogenesis in CRC. • Activation of the NF-κB pathway/Recruits bone marrow-derived immune cells for CRC development. • Inhibits NK cell and T cell activity/Promotes M2-like polarization of macrophages to promote tumor immune escape. • Increased levels of *Fusobacterium nucleatum* promoted microsatellite instability (MSI) with CpG island methylation phenotype (CIMP) in CRC. • Activation of the TLR4/AKT/Keap1/NRF2 signaling pathway increases the production of 12 and 13-EpOME and promotes CRC metastasis. • Activation of autophagy to induce chemoresistance.	Kostic et al., 2013; Tahara et al., 2014; Gur et al., 2015; Sivan et al., 2015; Wang and Huycke, 2015; Abed et al., 2016; Mima et al., 2017; Yu et al., 2017; Chen et al., 2018, 2020; Rubinstein et al., 2019; Hong et al., 2021; Hu et al., 2021; Zheng et al., 2021
*Escherichia coli*	Promote	• DNA damage • Increased IL-17c expression and inhibited tumor cell apoptosis by increasing bcl-2 and bcl-xl expression. • Pro-inflammatory infiltrate	Cuevas-Ramos et al., 2010; Song et al., 2014; Yu and Fang, 2015; Veziant et al., 2016; Bertocchi et al., 2021; Dalal et al., 2021; Lee et al., 2021
*ETBF*	Promote	• Release of BFT, leading to E-cadherin cleavage with intestinal epithelial cell detachment and disruption of the intestinal epithelial barrier. • Th17 immune response, promotes IL-6, TNF-α production, activates STAT3, NF-kB signaling pathway and promotes tumorigenesis. • DNA damage.	Destefano Shields et al., 2011; Goodwin et al., 2011; DeDecker et al., 2021
*Escherichia coli*	Promote	• It leads to CIN in epithelial progenitor cells, resulting in gene mutations.	Wang et al., 2015; Wang and Huycke, 2015

## 3 Application of postbiotics in colorectal cancer

**FIGURE 2:**
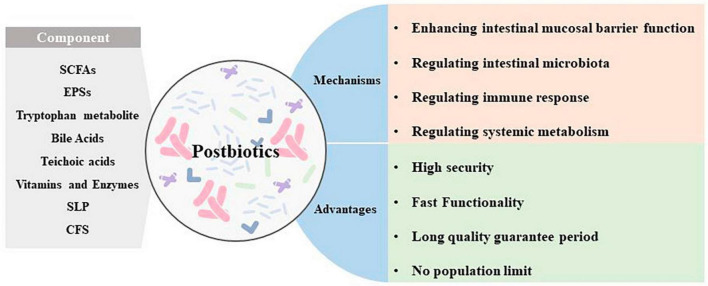
Main components, mechanism of action, and advantages of postbiotics.

**FIGURE 3:**
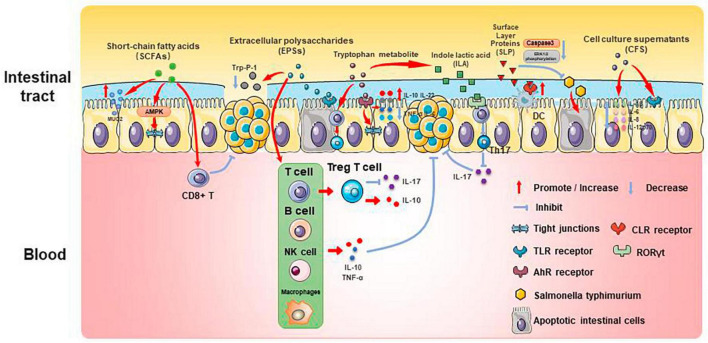
Mechanisms associated with postbiotic-related components protecting the gut and delaying CRC progression.

**FIGURE 4:**
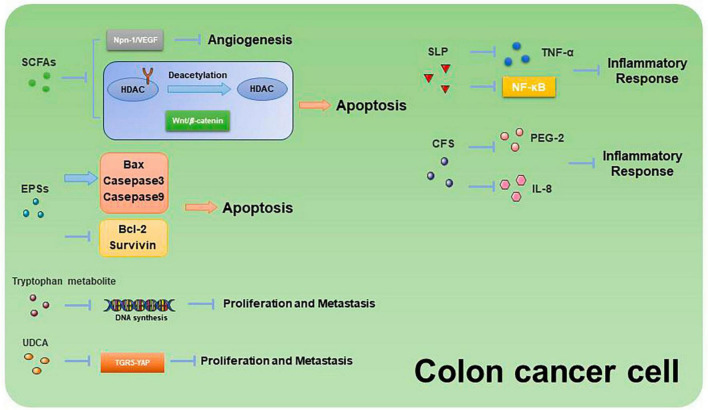
Mechanisms related to the promotion of apoptosis and inhibition of proliferation and metastasis in CRC cells by postbiotic-related components.

Past studies have revealed the use of microbial agents such as probiotics in CRC control. However, the biosafety of the products is somewhat controversial due to numerous uncertainties in the production, transportation and storage of these products. There is growing evidence that the health benefits of intestinal microbes may not require intact microbes, and that their inactivated by-products (including bacterial fragments and extracts) can still be useful. This is the category later associated with postbiotics. Postbiotics are preparations of inanimate microorganisms and their components that are beneficial to the host (Salminen et al., 2022). The main components of postbiotics include inactivated bacteria, bacterial fractions (cytosolic polypeptides, phosphoglycolic acids, intracellular and extracellular polysaccharides (EPSs), and surface proteins), and their metabolites (short-chain fatty acids (SCFAs), organic acids, bacteriocins, and enzymes) (Homayouni Rad et al., 2021). The mechanism of action of postbiotics to improve CRC progression is similar to that of prebiotics. It includes regulating intestinal microbiota, enhancing intestinal mucosal barrier function, regulating immune response and regulating systemic metabolism (Figure 2). However, compared to probiotics, postbiotics have a higher safety profile, better generalizability, longer shelf life and faster biological activity. The mechanism of improvement of CRC process by postnatal meta-related components is shown in Figures 3 and 4.

### 3.1 SCFAs

*Firmicutes*, *Bacteroides*, *Actinobacteria*, *Proteobacteria*, and *Verrucomicrobia* all create SCFAs, which can give host intestinal cells energy. SCFAs play an important role in gut microenvironmental homeostasis as essential components of the glycolytic process, act as protective molecules in the development of CRC, and change in response to changes in the composition of the intestinal microbiota. Therefore, SCFAs are closely associated with the development of CRC.

Butyric acid is produced by anaerobic bacteria in the intestinal tract, supplying energy to intestinal epithelial cells and inhibiting inflammatory responses and tumorigenesis. Butyric acid maintains the tight junctions of intestinal epithelial cells by activating the AMPK pathway and protects the intestinal mucosal barrier by inducing MUC2 secretion (Chen and Vitetta, 2021). Butyric acid regulates endogenous nervous system excitability and promotes intestinal motility, thereby preventing the development of CRC. Butyric acid also inhibits HDAC deacetylation and promotes apoptosis in CRC cells (Donohoe et al., 2014). In addition, butyric acid modulates the neuropilin-1/vascular endothelial growth factor pathway to inhibit CRC angiogenesis (Yu et al., 2010). It induces apoptosis in CRC tumor cells by inhibiting the Wnt/β-catenin signaling pathway (Wu et al., 2018). Butyric acid also promotes anti-tumor effects by modulating CD8 + T cell activity (He et al., 2021).

Acetic acid can be converted to butyric acid by bacteria such as *Roseburia* spp., *F. prausnitzii* and *Coprococcus* sp. to exert CRC inhibitory effects. Propionic acid can also act as an HDAC inhibitor to promote CRC apoptosis, but it is less effective than butyric acid. Formate secreted by *Fusobacterium nucleatum* can set off the AhR pathway, thus promoting CRC tumor invasion, and can serve as a tumor metabolite associated with CRC progression (Ryu et al., 2022; Ternes et al., 2022).

### 3.2 EPSs

Extracellular polysaccharides are bacterial surface macromolecules in the form of pods or pericellular mucus, which have a variety of biological functions such as immunomodulation, antitumor, and antioxidant. Previous studies have shown that EPSs inactivate the oncogenic component Trp-P-1 and alleviate the cancer process (Tsuda et al., 2008). In addition, EPSs can play an anti-inflammatory and anti-tumor role by enhancing the activity of T cells, B cells, NK cells and macrophages, promoting the expression of cytokines such as TNF-α and IL-10, and inducing apoptosis of tumor cells and scavenging of free radicals. For example, EPSs can inhibit the development of inflammation by binding to the TLR2 receptor of intestinal epithelial cells, inducing apoptosis and stimulating Treg T cells, inhibiting IL-17 production and promoting IL-10 expression (Levy et al., 2017; Zhou et al., 2017). In a study of the effect of EPSs on HT-29 proliferation, EPSs produced by *Lactobacillus lactis* promoted apoptosis and inhibited the proliferation of HT-29 cells by promoting the expression of Bax, Caspase3, and Caspase9, while decreasing the expression of Bcl-2 and Survivin genes (Tukenmez et al., 2019).

Extracellular polysaccharides can also affect the content of SCFAs to play an anti-inflammatory role. β-Glucan fermentation produces SCFAs, which activate macrophage Dectin-1 receptor, as well as T cells and NK cells, and enhances the immune response (Jayachandran et al., 2018; Chen and Li, 2020).

### 3.3 Tryptophan metabolite

Tryptophan is an essential amino acid that can be consumed from foods such as chicken, eggs, cheese and chocolate. It can be metabolized by bacteria (mainly *Lactobacillus reuteri* and *Clostridium sporogenes*) in the human intestinal tract to a variety of products such as 5-hydroxytryptamine (5-HT), kynurenine and indole, etc. These metabolites can be used as ligands for aromatic hydrocarbon receptors (AhR), and play an important role in the regulation of intestinal immune homeostasis, mucosal barrier function, inflammatory response and neural function.

Several studies have found that indole derivatives can inhibit inflammatory responses and improve the intestinal mucosal barrier by activating AhR, promoting the secretion of IL-22 and IL-10, decreasing the expression of TNF-α and IL-6, and increasing the production of tight junction proteins and mucins in intestinal epithelial tissue (Lanis et al., 2017; Shi et al., 2020). In addition, indole derivatives can reprogram CD4 + T cells in the intestinal epithelium to Treg T cells, street tumor immunity (Cervantes-Barragan et al., 2017). Another study showed that the tryptophan metabolic end product 8-hydroxyquinaldic acid could inhibit DNA synthesis and suppress proliferation and metastasis of colon cancer cells HT-29 and LS-180 (Walczak et al., 2020). A recent study of the chemopreventive effects of statins on CRC found that in a mouse model of CRC, atorvastatin inhibited tryptophan consumption by inhibiting the expression of indole derivatives in the intestinal epithelium, which in turn inhibited the consumption of tryptophan by intestinal epithelial cells, resulting in increased tryptophan concentration in the gut, increased abundance of *Lactobacillus reuteri*, whose catabolism of tryptophan produces the indole-3 -lactic acid (ILA) targets the transcription factor RORγt, which inhibits Th17 differentiation, decreases IL-17 expression, and inhibits CRC development (Han et al., 2023). This study demonstrates the role of tryptophan metabolites in mediating pharmacologic prevention of CRC.

### 3.4 Bile acids (BAs)

Primary bile acids include cholic acid (CA) and goose deoxycholic acid (CDCA), and there is increasing evidence that BA metabolism is strongly associated with CRC. CA and CDCA can promote CRC development by activating the NF-kB and JAK2/STAT3 pathways (Guan et al., 2022). In addition, a study showed that CA with CDCA was given to *N*-methyl-N′nitro-N-nitrosoguanidine-treated mice, which had a higher tumorigenicity than germ-free mice, demonstrating the critical role of BAs in CRC (Ridlon et al., 2016a).

Deoxycholic acid (DCA) is one of the secondary bile acids produced by *Clostridium*. DCA levels were found to be significantly elevated in CRC patients and correlated with intestinal mucosal hyperplasia. Studies have shown that the main mechanism by which DCA causes CRC is the activation of COX-2 and lipoxygenase, which catabolizes arachidonic acid to produce prostaglandins and ROS, triggering inflammation, angiogenesis, DNA damage, and inhibition of DNA damage repair (Cai et al., 2022). In addition, DCA stimulates the ERK signaling pathway and regulates p53 expression, thereby promoting the development of CRC.

Lithocholic acid (LCA) is another secondary bile acid produced by *Clostridium*. Similar to DCA, LCA can induce CRC by damaging the intestinal mucosa through the production of ROS, reducing apoptosis, enhancing cell proliferation, contributing to DNA damage, and stimulating inflammatory responses (Sinha et al., 2020). In addition, LCA regulates muscarinic 3 receptor and Wnt/β-catenin pathways to promote tumor stem cells in CRC (Farhana et al., 2016). LCA also induced the expression of MMP-1, MMP-2, and MMP-7 genes and stimulated the urokinase plasminogen activator receptor, promoting invasion and metastasis of CRC cells (Rossi et al., 2020). Another study found that LCA elevated the expression of the cytokine IL-8, which activated the ERK/MAPK signaling pathway, thereby inhibiting STAT3, stimulating CRC angiogenesis, and promoting CRC development (Nguyen et al., 2017).

Ursodeoxycholic acid (UDCA) has been reported to be protective against digestive disorders. However, its effect on CRC remains controversial. Studies have shown that UDCA can play a therapeutic role in reducing intestinal inflammation by modulating the epidermal growth factor receptor/ERK pathway and reducing harmful secondary bile acids (DCA and LCA) (Ridlon et al., 2016b). In addition, UDCA inhibited the activation of COX-2 by DCA and suppressed CRC progression through the TGR5-YAP pathway (Zhang et al., 2021). However, it has also been reported that UDCA can be converted by microorganisms into harmful secondary bile acids that promote the development of CRC. Meanwhile, the study found no significant effect of UCDA in reducing cancer risk (Sorbara and Pamer, 2022). These studies suggest that modulation of bile acid production, especially DCA with LCA, may have a positive effect on the prevention and treatment of CRC.

### 3.5 Teichoic acids (TA)

Teichoic acids (TA) are the main components of the cell walls of Gram-positive bacteria, and studies have shown that TA and lipoteichoic acid (LTA), obtained by extraction with butanol and phenol, can mediate immune responses by inhibiting the production of certain inflammatory factors (e.g., IL-12 and IL-10) (Kaji et al., 2010) and modulating the function of Treg T cells to suppress intestinal inflammation and maintains intestinal homeostasis, and exerts antitumor and antioxidant effects (Tomkovich and Jobin, 2016). However, some studies have found that LTA not only fails to reduce the inflammatory response, but also causes damage to the intestinal mucosa (Zadeh et al., 2012). Therefore, further studies on the protective effects of TA and LTA on the intestinal tract are needed.

### 3.6 Vitamins and enzymes

In addition to obtaining vitamins from food, animals synthesize some vitamins through gut microbes, which play an important role in the storage and conversion of vitamins in the body. Vitamin D can regulate intestinal immunity by influencing T cell activation. Vitamin A plays an important role in the functional integrity of the cuprocytes. Deficiency of vitamin A and its receptors can lead to disruption of the intestinal mucosal barrier and disruption of the intestinal immune system, increasing the risk of intestinal infections and injuries.

Gut microbes also produce antioxidant enzymes such as glutathione peroxidase (GPX), superoxide dismutase (SOD), catalase and reduced coenzyme I oxidase to protect against oxidative damage caused by ROS.

### 3.7 SLP

Surface Layer Proteins (SLP) are bioactive macromolecules encapsulated on the surface of the cell wall of many bacteria and archaea, and are involved in the regulation of various cellular physiological and biochemical processes. Studies have shown that SLP from lactic acid bacteria modulate intestinal inflammation by resisting the adhesion and invasion of pathogenic bacteria, regulating Th1 and Th17 activities, and influencing the immune response.

Normal apoptosis mediates clearance of invading bacteria and facilitates repair of the intestinal epithelial barrier. In contrast, abnormal apoptosis induced by pathogenic bacteria leads to inflammation. SLP of various *Lactobacillus* species play important roles in anti-inflammatory and immunomodulatory aspects. SLP of *Lactobacillus acidophilus* antagonizes Salmonella typhimurium-induced apoptosis by inhibiting caspase-3. SLP of *Lactobacillus acidophilus* ATCC-4356 induced cell proliferation and differentiation by inhibiting Salmonella typhimurium-induced apoptosis in Caco-2 cells and decreasing ERK1/2 phosphorylation (Li et al., 2011). In addition, SLP of *Lactobacillus acidophilus* NCFM binds to C-type lectin receptors (CLRs) on the surface of dendritic cells, regulates intestinal immunity, enhances intestinal barrier function, and slows down inflammation (Konstantinov et al., 2008).

Surface Layer Proteins isolated from Propionibacterium fischeri reduced TNF-α levels in HT-29 cells (Rabah et al., 2018). SLP isolated from *Lactobacillus helveticus* MMLh5 inhibited the NF-κB pathway in Caco-2 cells and exerted anti-inflammatory effects (Taverniti et al., 2013). SLP from *Lactobacillus plantarum* reversed pathogenic E. coli-induced intestinal epithelial cell damage (Liu et al., 2011).

### 3.8 CFS

Probiotic cell culture supernatants contain organic acids, short-chain fatty acids, bacteriocins, and other active substances, and studies have shown that the probiotic CFS has a health-promoting effect on human health. In a study in which dendritic cells attacked by E. coli were treated with *Lactobacillus rhamnosus* and its CFS, the results showed a significant decrease in the levels of pro-inflammatory factors (IL-1β, IL-6, IL-8, and IL-12p70) in CFS-treated cells as compared to *Lactobacillus rhamnosus*, indicating that in the presence of E. coli CFS is more effective than probiotics in reducing the secretion of pro-inflammatory factors (Bermudez-Brito et al., 2014). CFS of *Bifidobacterium shortum* CNCM?-4035 down-regulates pro-inflammatory pathways by reducing pro-inflammatory factors and chemokines in dendritic cells attacked by Salmonella typhi through activation of the TLR and protects the body against highly infectious pathogens such as Salmonella typhi (Bermudez-Brito et al., 2013). CFS of probiotics such as *Lactobacillus acidophilus*, *Lactobacillus casei*, *Lactococcus lactis*, *Lactobacillus rohita*, and *Saccharomyces boulardii* down-regulate the expression of PGE-2 and IL-8 in HT-29, as well as regulating the expression of IL-1β, IL-6, TNF-α, and IL-10 in macrophages, exerting anti-inflammatory effects (De Marco et al., 2018). In addition, CFS of *Lactobacillus plantarum* not only reverses the resistance of tumor cells to 5-FU and reduces CRC stem cells, but also exerts specific antiproliferative and apoptosis-inducing effects on tumor cells, while having no effect on normal cells (Chuah et al., 2019). This suggests that CFS of *Lactobacillus plantarum* can be used as a complementary and adjuvant therapy for CRC.

## 4 Advantages

Compared with traditional probiotics, NGP and FMT, which regulate intestinal microbiota and protect intestinal function, postbiotics have higher safety, faster functionality, longer quality guarantee period and no population limit, etc. First of all, postbiotics are the bacterial components and bacterial metabolites obtained by non-heat inactivation technology, with a clear chemical structure and composition, so it can avoid the serious safety hazards of traditional probiotics therapy such as bacterial translocation, increasing the host’s resistance to antibiotics, transmission of drug-resistant genes or contamination of pathogenic bacteria, etc., and it has a higher level of safety. Secondly, traditional probiotics therapy often need to pass through gastric acid and bile salts, colonize in the intestines and finally participate in intestinal metabolism before they can exert their biological activities. As a direct metabolite of intestinal microbiota, probiotics can be directly absorbed by the intestines after drinking and participate in intestinal metabolism to exert their biological activity, which has a faster onset of action. During the production and storage of traditional probiotics, the characteristics of the bacteria themselves and the environment (including temperature, humidity, pressure and oxygen content, etc.) may lead to a reduction in the biological activity or death of the bacteria, thus affecting the quality and efficacy of the product, and may even lead to adverse reactions. In fact, there have been reports of discrepancies between the actual probiotic content and the standardized content of commercial probiotic products. Postbiotics, on the other hand, after being processed by inactivation technology, exhibit heat-resistant, acid-resistant and durable properties, and are not easily interfered with by antibiotics and other substances, providing a Longer and more stable shelf life with higher therapeutic benefit. Finally, for special groups such as children and sensitive people, postbiotics do not have the risks associated with the application of traditional probiotics, and there is no restriction on the number of people, so they have a wider range of applications.

However, of the 7 randomized controlled trials of 1,740 children younger than 5 years of age, 3 assessed adverse reactions in subjects. In 1 trial investigating *Lactobacillus*-origin postbiotics on acute diarrhea in children, 36 of the 40 children in the trial group experienced symptomatic relief, 1 experienced an adverse reaction (severe dehydration), and the remaining 2 studies showed no adverse reactions in subjects (Malagón-Rojas et al., 2020). This suggests that there may still be some adverse reactions to postbiotic, but the incidence is low. Subsequent studies should pay attention to improving the handling and preservation of postbiotic products, improving the safety of postbiotic products, and decreasing the incidence of adverse reactions to postbiotic products.

## 5 Conclusion and outlook

Intestinal microbiota and its metabolites are closely related to the progression of CRC. Intestinal microbiota can influence the development of CRC by altering the intestinal microenvironmental homeostasis, influencing the intestinal immune response, and secreting a variety of metabolites. Since certain bacteria are more likely to affect the development of CRC, together with the differences in the composition and abundance of the flora in CRC patients have been reported in this study.

More and more microbial agents protect the intestinal barrier function by regulating the composition and function of intestinal microorganisms, and Postbiotic stands out among many microbial agents due to its unique safety and stability. Numerous studies have shown that postbiotics exert anti-tumor effects by regulating the composition of intestinal microorganisms, immunomodulation, inhibiting proliferation and inducing apoptosis, enhancing the intestinal mucosal barrier, and regulating systemic metabolism. Therefore, the prospect of postbiotic as an adjuvant and supplemental agent for tumor treatment is wide-ranging. In addition, certain postnatal metabolic components, such as DCA, are significantly elevated in CRC patients and can be used as an indicator for early diagnosis and screening for CRC.

Despite the unique advantages of postbiotics in microbial preparations, there are still many challenges in the application of postbiotics in the prevention and treatment of CRC. Firstly, the complexity of the components and the non-uniformity of the production process of postbiotic formulations have led to some ambiguity in the definitional scope of postbiotics; therefore, more in-depth studies are needed to investigate the mechanism of action of postbiotic-related components and to determine a reasonable range of components as well as a uniform production process. Secondly, in the research and development of postbiotics, it is recommended to prioritize the selection of microorganisms that have completed safety evaluations and the safety evaluations of the prepared postbiotic components, rather than relying solely on the safety of the original strains of bacteria used in the preparation of postbiotics to judge the safety of postbiotics. Thirdly, traditional probiotics have clear dosage standards, while the qualitative and quantitative analysis methods have not been established for postbiotics, and there are certain problems in the quality regulation and use of postbiotics, so it is necessary to construct a quantitative and effective relationship evaluation method of the role of postbiotics to provide theoretical support for the development of the postbiotics industry. In addition, the interaction with food components should be considered during the use of postbiotic elements. Finally, although recent studies on postbiotics have demonstrated the potential for CRC prevention and treatment, the related mechanism of action still requires further research, and there is still a lack of more clinical studies on the effective treatment of CRC. In conclusion, as an emerging field, the advantages and potential of postbiotics in CRC diagnosis and treatment still deserve attention and expectation.

## Author contributions

WX: Writing – original draft, Writing – review and editing. Y-SZ: Funding acquisition, Writing – review and editing. X-JL: Writing – original draft, Writing – review and editing. Y-KK: Writing – original draft, Writing – review and editing. Q-YP: Writing – review and editing. H-ZY: Funding acquisition, Writing – review and editing.
